# Ketogenic Diet–Associated Remodeling of the Cortical Proteome in Rats With Genetic Absence Epilepsy

**DOI:** 10.1002/brb3.71757

**Published:** 2026-10-07

**Authors:** Aslihan Gunel, Ulku Guler, Aylin Toplu, Nazli Busra Acikgoz, Bora Onat, Ariorad Moniri, Bekir Salih, Osman Ugur Sezerman, Filiz Onat

**Affiliations:** ^1^ Department of Neuroscience Graduate School of Health Sciences, Acibadem Mehmet Ali Aydinlar University Istanbul Türkiye; ^2^ Department of Molecular Biology and Genetics, Faculty of Science and Arts Kırsehir Ahi Evran University Kirsehir Türkiye; ^3^ Department of Chemistry‐Biochemistry, Faculty of Science and Arts Kırsehir Ahi Evran University Kirsehir Türkiye; ^4^ Department of Chemistry Faculty of Science Hacettepe University Ankara Türkiye; ^5^ School of Medicine, Department of Medical Pharmacology Health Science University Istanbul Türkiye; ^6^ Department of Biotechnology Ankara University Ankara Türkiye; ^7^ School of Medicine Acibadem Mehmet Ali Aydinlar University Istanbul Türkiye; ^8^ Department of Biostatistics and Medical Informatics School of Medicine Acibadem Mehmet Ali Aydinlar University Istanbul Türkiye; ^9^ Department of Medical Pharmacology School of Medicine Acibadem Mehmet Ali Aydinlar University Istanbul Türkiye

**Keywords:** ketogenic diet, absence epilepsy, proteomics, somatosensory cortex, GAERS

## Abstract

**Purpose:**

This study investigated the effects of ketogenic diet (KD) intervention on the cortical proteome in genetic absence epilepsy rats from Strasbourg (GAERS), a well‐established model of absence epilepsy. The ketogenic diet is a therapeutic intervention for epilepsy, yet its mechanisms remain incompletely understood.

**Method:**

Thirty‐day‐old male GAERS rats were fed a KD (88.8% fat, 10.2% protein, 1% carbohydrate; *n* = 4) or a normal diet (ND; *n* = 3), with Wistar rats (*n* = 3) as healthy controls. A shotgun proteomics approach with data‐dependent acquisition (DDA) was used to analyze the somatosensory cortex (SCx), a key cortical driver of absence seizures. Data were processed with MaxQuant and Perseus.

**Findings:**

Compared to Wistars, GAERS‐ND rats showed significant alterations in 526 proteins in SCx and KD treatment significantly altered 121 proteins in SCx of GAERS. Notably, KD was associated with partial shifts in the expression patterns of 22 proteins toward those observed in Wistar controls. Most KD‐affected proteins were synaptic (72% SCx) and mitochondrial. Pathway analysis indicated that ketogenic diet–associated protein changes were enriched in axon guidance, neurodevelopment, and metabolic pathways in the somatosensory cortex.

**Conclusion:**

Together, these results suggest that synaptic and mitochondrial protein alterations in this region are associated with cortical molecular adaptations observed under ketogenic diet administration in absence epilepsy.

## Graphical abstract

Proteomic profiling of the somatosensory cortex revealed that ketogenic diet was associated with remodeling of cortical protein networks in GAERS absence epilepsy, with altered proteins enriched in synaptic and mitochondrial pathways.

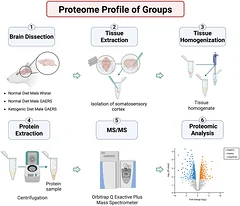

## 1 Introduction

Absence epilepsy, a type of epilepsy accounting for 10%–17% of cases in children and adolescents, is characterized by abrupt cessation of voluntary motor activity and 3–4 Hz generalized spike‐and‐slow wave discharges (SWDs) on EEG (Scheffer et al. 2017). Despite available anti‐seizure medications (ASM), a subset of patients continues to experience seizures, classified as drug‐resistant epilepsy (Pluta and Jablonski 2011).

The ketogenic diet (KD) has been used to treat epilepsy since 1911 in modern medicine (Guelpa 1911). While its use declined with new ASM development, recent studies suggest that it may contribute to seizure reduction in selected cases of absence epilepsy (Wheless 2008). However, the precise mechanisms underlying the ketogenic diet's anticonvulsant effects in absence epilepsy remain unclear. A limited number of experimental and clinical studies have also explored the potential effects of the ketogenic diet on seizure control in absence epilepsy (Groomes et al. 2011). Clinical studies, however, face limitations and animal models provide controlled environments for research. Metabolically, this dietary approach promotes heightened gluconeogenesis and ketogenesis, altered mitochondrial activity, neurotransmitter homeostasis, and ion channel behavior (Youngson et al. 2017). The genetic absence epilepsy rats from Strasbourg (GAERS) (Depaulis et al. 2016) is a widely used model, exhibiting spontaneous 7–11 Hz SWDs, valuable for investigating absence epilepsy pathophysiology and treatments.

Proteomics, the study of the entire protein complement, offers a powerful approach to identify pathological disease mechanisms in neurological disorders (Aebersold et al. 2018). Proteomic tools are increasingly used to identify differentially expressed proteins and proteoforms, providing insights into dynamic processes within interacting proteins and biological networks involved in epilepsy (Scott 2016). Recent advancements in proteomics technologies provide promising avenues for exploring the molecular mechanisms underlying the effects of the ketogenic diet (Leitner et al. 2023; Zheng et al. 2020).

Given the increasing interest in the ketogenic diet as a therapeutic strategy, elucidating its molecular effects in absence epilepsy is essential—particularly at the regional level. While proteomic approaches have been employed in other epilepsy models (Leitner et al. 2023; Zheng et al. 2020), to our knowledge, no study has yet examined how the ketogenic diet influences the brain proteome in genetic models of absence epilepsy. Although absence epilepsy is commonly managed with anti‐seizure medications, pharmacological treatment does not always provide optimal or sustained seizure control and may be limited by tolerability over time. In addition, current therapies largely focus on controlling clinical symptoms and offer limited insight into the molecular‐ and network‐level changes associated with the disorder. For this reason, non‐pharmacological approaches such as the ketogenic diet remain of interest, particularly for exploring complementary mechanisms that cannot be addressed by drug‐based strategies alone. Accordingly, the present study does not seek to propose the ketogenic diet as a replacement for established treatments, but rather to examine its effects on seizure‐related cortical pathways at the proteomic level.

The GAERS normal diet and GAERS ketogenic diet groups included in the present study were derived from the same experimental cohort used in our previous metabolic and EEG study (Acikgoz et al. 2026). In that study, ketogenic diet treatment increased β‐hydroxybutyrate levels while reducing blood glucose, body weight gain, and the mean duration of individual spike‐and‐wave discharges, whereas seizure number and cumulative seizure duration remained unchanged. The present study examined cortical proteomic changes in the same GAERS cohort to complement these previously reported physiological findings. A Wistar control group was included only in the present proteomic study.

In this study, we investigated the impact of the ketogenic diet on absence seizures in GAERS rats by integrating high‐throughput shotgun proteomics analyses. Absence seizures originate in the somatosensory cortex (SCx) and spread through cortico‐thalamo‐cortical networks (D'Arcangelo et al. 2002). Therefore, we focused our proteomic analysis on the somatosensory cortex and compared protein expression profiles among control Wistar rats, GAERS, and GAERS receiving the ketogenic diet from postnatal Day 30 until decapitation at postnatal Day 72. This approach allowed us to identify key molecular pathways associated with both epilepsy and dietary intervention, providing system‐level insight into cortical molecular responses under ketogenic diet administration in absence epilepsy.

## 2 Materials and Methods

All chemicals were obtained from Sigma Aldrich, and Trypsin‐LysC from Promega.

### 2.1 Experimental Animals

GAERS rats (Postnatal 30‐day‐old male) were divided into two groups: a normal diet group (*n* = 3) and a ketogenic diet group (*n* = 4). Three Wistar rats served as healthy controls. The study, conducted in compliance with ethical guidelines at Marmara University School of Medicine's Department of Medical Pharmacology, maintained animals on a 12‐h light/dark cycle at 22°C–24°C. The control group continued on a normal diet, while the experimental group received a ketogenic diet, both ad libitum. Dietary interventions were maintained until the day of sacrifice. Animals were housed individually and weighed weekly. Ethical approval was granted by the Marmara University School of Medicine (approval number: 41.2022.mar). The GAERS normal diet and GAERS ketogenic diet groups analyzed in the present study were obtained from the same experimental cohort previously used for metabolic and EEG analyses (Acikgoz et al. 2026). A Wistar control group was included specifically for the present proteomic study.

This study is reported in accordance with the ARRIVE 2.0 guidelines (Percie du Sert et al., 2020).

### 2.2 Diet Therapy

As described by Acikgoz et al. (2026), diet therapy was implemented with both groups initially receiving a normal diet (56% carbohydrates, 30% protein, and 14% fat) until Day 30. Subsequently, the experimental group transitioned to a ketogenic diet composed of 88.8% fat, 10.2% protein, and 1% carbohydrate. The diets, supplied by Arden Research (Ankara, Türkiye), were stored at −20°C, with daily portions kept at +4°C until use. Water was available ad libitum throughout the study.

### 2.3 Proteomic Analysis

#### 2.3.1 Isolation of the Tissue

Ten male rats (four ketogenic‐fed GAERS, three normal diet‐fed GAERS, and three healthy normal diet‐fed Wistars) were euthanized by decapitation under isoflurane (1%–3% isoflurane in O_2_) anesthesia. The somatosensory cortex was dissected in cold 1x PBS with protease and phosphatase inhibitors and stored at −80°C. For total protein extraction, the Weiss protocol (Weiss et al. 2012), was employed. In this procedure, tissues were homogenized using a lysis buffer (10% w/v) that contained 150 mM NaCl, 50 mM Tris‐HCl (pH 7.5), 1% Nonidet P‐40, and a full complement of protease and phosphatase inhibitors. Total protein concentration was determined using the bicinconinic acid assay (BCA) (Smith et al. 1985).

Each cortical sample was processed and analyzed individually. No samples were pooled during homogenization, protein extraction, or LC‐MS/MS analysis.

### 2.4 Randomization and Sample Handling

Animals were randomly assigned to the experimental groups. Investigators responsible for animal dissection and sample collection were aware of the experimental group identities. Following tissue collection, samples were assigned numerical codes, and investigators involved in protein extraction and LC‐MS/MS analysis were blinded to the experimental group identities. Group identities were subsequently revealed during MaxQuant data processing for comparative analysis in MaxQuant and Perseus. No samples were excluded after group allocation or during proteomic analysis.

#### 2.4.1 In Solution, Protein Digestion

Each sample (100 µg protein) was transferred to microtubes and reduced with dithiothreitol (DTT, 5 mM), and alkylated with iodoacetamide (IAA, 50 mM) for 1 h at room temperature in the dark. Excess IAA was quenched with DTT (10 mM). Proteins were precipitated using methanol/chloroform, and the precipitate was dissolved in 8 M urea, 50 mM Tris‐HCl (pH 8.5). The solution was then diluted with Tris‐HCl buffer to a final concentration of 1 M urea and subjected to overnight tryptic digestion (enzyme‐to‐protein ratio 1:100, w/w) at 25°C. Digestion was terminated by the addition of trifluoroacetic acid (TFA, 0.5% v/v). Peptides were purified using Sep‐Pak cartridges, dried, and reconstituted in Milli‐Q water (18.2 MΩ·cm) to a final concentration of 1 µg/µL (Saei et al. 2019).

#### 2.4.2 Mass Spectrometry

Peptides were separated on a 50 cm EASY‐Spray C18 column (75 µm ID, 2 µm particles, 100 Å) connected to an Ultimate 3000 RSLnano system (Dionex, Thermo Scientific) and analyzed on a Q Exactive Plus mass spectrometer. Samples were loaded with buffer A (0.1% formic acid in water) and eluted over 150 min with a 5%–95% gradient of buffer B (95% ACN, 0.1% FA) at 250 nL/min. MS spectra were acquired in data‐dependent mode with a 500–2500 m/z range. Peptide fragmentation was performed with HCD at 29 NCE, and MS/MS spectra were recorded at 17,500 resolution.

#### 2.4.3 Data Analysis

MaxQuant (v1.6) was used for protein identification and label‐free quantification (LFQ) (Cox and Mann 2008; Cox et al. 2011). The search parameters included 20 ppm and 4.5 ppm mass accuracy for the first and main searches, respectively, up to two missed cleavages, and peptide charges of 2–7. Fixed and variable modifications were set as carbamidomethyl (Cys), oxidation (Met), and acetylation (N‐terminus). The raw data were queried against *Rattus novergicus* protein sequences (UniProt: UP000002494) with a 1% FDR. Perseus (v1.7) (Tyanova et al. 2016) was used for data analysis, with LFQ values log_2_‐transformed and filtered. The criteria for accepting LFQ values was a minimum of two valid measurements in at least one of the three experimental groups. Imputation was performed conservatively using the lowest observed values to minimize artificial inflation of protein abundance estimates. Welch's *t*‐tests were performed to identify significant differences between GAERS versus Wistar and GAERS_ketogenic diet_ versus GAERS_normal diet_. To control for multiple testing Benjamini–Hochberg false discovery rate (FDR) correction (FDR = 0.05) was subsequently applied in Perseus, and *q*‐values were calculated (Benjamini and Hochberg 1995). Protein abundance changes are reported as log_2_ fold change (log_2_FC), representing both the magnitude and direction of differential protein expression.

#### 2.4.4 Principal Component Analysis

Proteomic LFQ data were analyzed in Python 3.13.6 using pandas, scikit‐learn, and matplotlib. Region‐specific matrices (somatosensory cortex) were prepared by selecting predefined LFQ columns. Data were standardized and subjected to PCA (5 components), with variance explained reported. PCA results were visualized as pair plots (5 × 5 grids) with group‐specific colors/markers.

### 2.5 Bioinformatics Analysis

#### 2.5.1 Synaptic Protein Enrichment and Prediction of Subcellular Localization

To evaluate whether differentially expressed proteins were enriched in synaptic regions, the SynGO database and a recent synaptic proteome reference in mouse brain tissue were utilized (Koopmans et al. 2019; van Oostrum et al. 2023). Subcellular localization of proteins was predicted using DeepLoc v2.1 (Ødum et al. 2024). The significance of synaptic enrichment and compartment‐specific distribution was assessed via hypergeometric analysis.

#### 2.5.2 Protein–Protein Interaction and Pathway Enrichment

Protein‐protein interaction and pathway enrichment analyses were conducted using ZS‐Revelen (Li et al. 2017) with a significance threshold of *p* ≤ 0.05. Reactome was used as the primary reference for pathway data. Additionally, epilepsy‐ and seizure‐associated protein enrichment was performed using DisGeNET (via the Enrichr tool) and DOID (via ZS‐Revelen) (Li et al. 2017).

## 3 Results

### 3.1 Impact of KD on Measured Metabolic Parameters

As previously reported (Acikgoz et al. 2026) ketogenic diet treatment increased β‐hydroxybutyrate levels and reduced blood glucose and body weight gain.

### 3.2 Proteomics

In total, 2079 proteins were identified and quantified in our proteomics analyses. After applying a quality control filter requiring at least two valid LFQ measurements in one of the experimental groups, 1881 proteins were retained for further analysis.

In the GAERS versus Wistar comparison, 526 proteins in SCx were significantly altered after Benjamini–Hochberg correction (FDR < 0.05). Of these, 455 in SCx (439 upregulated, 16 downregulated) also met the |log_2_FC| ≥ 2 criterion (Figure 1A; Table S1).

**FIGURE 1:**
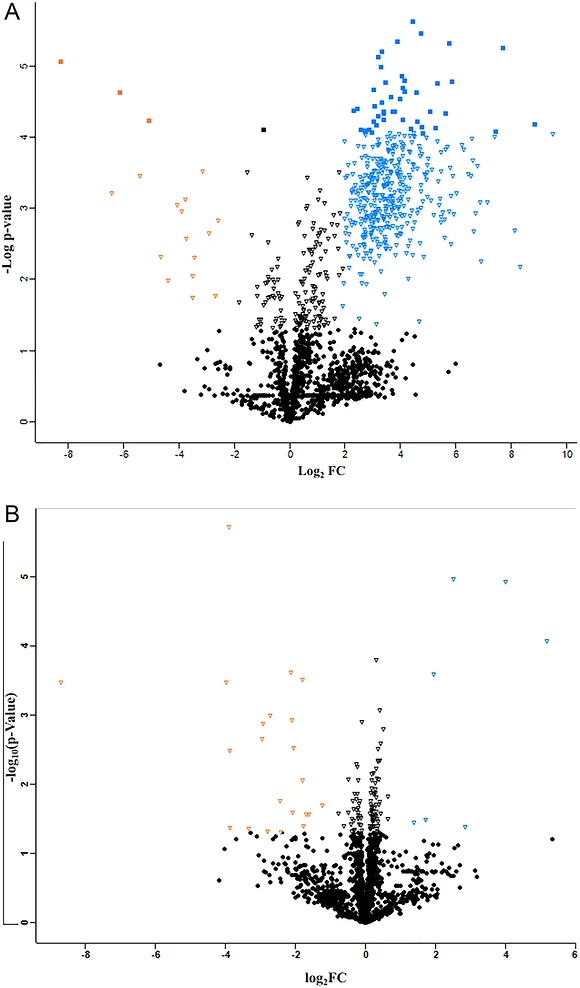
Volcano plots showing differential protein expression in the somatosensory cortex (SCx). (A) GAERS versus Wistar rats. The x‐axis represents log_2_ fold change, and the y‐axis shows −log_10_(*p‐*value) from Welch's *t*‐test. Proteins with statistically significant differential expression (*p* < 0.05) and |log_2_FC| ≥ 2 are shown as blue triangles (upregulated in GAERS) and orange triangles (downregulated in GAERS). Statistically significant proteins with |log_2_FC| < 2 are shown in black triangles. Nonsignificant proteins (*p* ≥ 0.05) are displayed as filled black circles. Filled orange and blue squares indicate the top 50 proteins ranked by statistical significance after Benjamini–Hochberg correction (FDR‐adjusted *p*‐value) among proteins with |log_2_FC| ≥ 2, whereas the remaining proteins meeting the same criteria are displayed as open triangles. (B) GAERS ketogenic diet versus GAERS normal diet rats. The x‐axis represents log_2_ fold change, and the y‐axis shows −log_10_(*p‐*value) from Welch's *t*‐test. Proteins with statistically significant differential expression (*p* < 0.05) and |log_2_FC| ≥ 2 are shown as blue triangles (upregulated) and orange triangles (downregulated). Statistically significant proteins with |log_2_FC| < 2 (log_2_FC between −2 and +2) are shown in black triangles, while nonsignificant proteins (*p* ≥ 0.05) are displayed as filled black circles.

Upon comparing GAERS rats fed a ketogenic diet with those on a normal diet, 121 proteins were significantly altered in SCx (66 upregulated, 55 downregulated) (Figure 1B; Table S2). To highlight the most statistically robust changes, the top 20 proteins ranked by *p*‐value are shown in Table 1.

**TABLE 1:** Top 20 differentially expressed proteins following ketogenic diet treatment in Scx of GAERS rats, ranked by *p*‐value.

UniProt ID	Protein name	Gene symbol	log_2_FC	−log_10_(*p*‐value)	Welch's *t*‐test *q*‐value (Benjamini–Hochberg adjusted *p*‐value)
B2RZ78	Vacuolar protein sorting‐associated protein 29	Vps29	−3.885849635	5.70804952	0.00368
B2DD29	Serine/threonine‐protein kinase BRSK1	Brsk1	2.522530556	4.962024118	0.00751
A0A0H2UHY3	Limbic system‐associated membrane protein	Lsamp	4.000090599	4.921463835	0.00751
B0BNK1	RAB5C. member RAS oncogene family	Rab5c	5.189702034	4.057037737	0.04124
Q9JHL4	Drebrin‐like protein	Dbnl	0.316766103	3.793242898	0.06056
B0BNM7	Sco1 protein	Sco1	−2.114095052	3.601013906	0.06406
D3Z981	Plexin A1	Plxna1	1.96157074	3.579918082	0.06406
A0A0G2K3K2	Actin	Actb	−8.673481623	3.469826584	0.06406
P97615	Thioredoxin	Txn2	−3.953560511	3.467781939	0.06406
A0A0H2UHM0	Vacuolar protein sorting‐associated protein 28 homolog	Vps28	−1.791840871	3.497573891	0.06406
A0A0G2K330	Trifunctional enzyme subunit beta	Hadhb	0.427258968	3.067923269	0.14624
Q9JKS6	Protein piccolo	Pclo	−2.717306773	2.977900078	0.16493
D4A383	Protein tweety homolog	Ttyh3	−2.090946833	2.919372532	0.17149
Q7HKW3	NADH‐ubiquinone oxidoreductase chain 4	Mtnd4	−2.919536591	2.864057593	0.17149
Q8R491	EH domain‐containing protein 3	Ehd3	−0.088922819	2.888962592	0.17149
P21775	3‐ketoacyl‐CoA thiolase A	Acaa1b	0.514816443	2.793960505	0.18893
D3ZI60	60S ribosomal protein L27a	Rpl27a	−2.936788559	2.641935225	0.25235
O54861	Sortilin	Sort1	0.248581092	1.372722474	0.67666
B5DEN5	Elongation factor 1‐beta	Eef1b2	0.363298893	2.508896829	0.29138
M0RC17	Cell adhesion molecule L1‐like	Chl1	−2.036486308	2.515420474	0.29138

*Note*: log_2_FC values indicate the magnitude and direction of protein abundance changes between the compared groups, and *q*‐values were calculated using the Benjamini–Hochberg method. Proteins are ranked based on –log_10_(*p*‐values). The complete list of statistically significant proteins is provided in Table S2.

Notably, ketogenic diet administration was associated with shifts in the expression of multiple proteins altered in GAERS rats. In the somatosensory cortex, the dietary intervention partially shifted the expression of 22 proteins toward the expression patterns observed in Wistar controls. These proteins had been significantly altered (|log_2_FC| ≥ 2) in the GAERS versus Wistar comparison (Table 2). In addition, a subset of proteins was significantly affected in both the GAERS versus Wistar and GAERS ketogenic diet versus GAERS normal diet comparisons, and these shared proteins are summarized in Table S3.

**TABLE 2:** Proteins showing expression shifts following ketogenic diet administration in the somatosensory cortex of GAERS rats.

log_2_FC in ND	Uniprot ID	Protein name	Gene symbol	log_2_FC in KD
3.651	P28037	Cytosolic 10‐formyltetrahydrofolate dehydrogenase	Aldh1l1	−2.775
5.865	B2RZ72	Actin‐related protein 2/3 complex subunit 4	Arpc4	−0.202
2.141	P49088	Asparagine synthetase [glutamine‐hydrolyzing]	Asns	−1.685
2.281	F1LQ09	Atlastin GTPase	Atl2	−1.766
2.036	M0RC17	Cell adhesion molecule L1‐like	Chl1	−2.036
−1.541	P08082	Clathrin light chain B	Cltb	0.447
1.782	Q5XIA5	Coenzyme A synthase	Coasy	−1.782
3.2	A0A0G2JSP3	Cullin‐3	Cul3	−0.334
1.792	Q5XIP0	DnaJ (Hsp40) homolog	Dnajb4	−1.792
0.528	P05708	Hexokinase‐1	Hk1	−0.125
2.952	P55067	Neurocan core protein	Ncan	−2.432
5.756	D3ZNW5	Neurofascin	Nfasc	−0.251
2.685	Q6AXT5	Ras‐related protein Rab‐21	Rab21	−0.37
2.937	D3ZI60	60S ribosomal protein L27a	Rpl27a	−2.937
2.114	B0BNM7	Sco1 protein	Sco1	−2.114
4.999	A0A0G2JYF0	Sodium‐driven chloride bicarbonate exchanger	Slc4a10	−0.335
2.027	E9PU13	Sorting nexin 4	Snx4	−1.61
2.591	Q6PCT3	Tumor protein D54	Tpd52l2	−2.077
3.859	Q9JJW1	Tetraspanin‐2	Tspan2	−3.859
2.091	D4A383	Protein tweety homolog	Ttyh3	−2.091
3.954	P97615	Thioredoxin	Txn2	−3.954
1.792	A0A0H2UHM0	Vacuolar protein sorting‐associated protein 28 homolog	Vps28	−1.792

*Note*: log_2_FC values indicate the magnitude and direction of protein abundance changes between the compared groups.Abbreviations: FC, fold change; GAERS, genetic absence epilepsy rats from Strasbourg; KD, ketogenic diet; ND, normal diet; SCx, somatosensory cortex.

### 3.3 Bioinformatics Analysis

#### 3.3.1 Synaptic Protein Enrichment and Subcellular Localization Prediction

To determine whether altered proteins were enriched in synaptic compartments, human orthologs were queried using the SynGO database (Koopmans et al. 2019) and a recent reference dataset by van Oostrum et al. (2023). Proteomic analysis demonstrated that synaptic proteins played a dominant role among those differentially expressed, under both normal and ketogenic diet conditions. In the comparison between GAERS and Wistar rats on a normal diet, 65% (SCx) of the differentially expressed proteins were synaptic. When examining the effects of the ketogenic diet on the GAERS model, this synaptic enrichment became even more pronounced, with the proportion rising to 72% in the SCx. The statistical significance of these high proportions was confirmed by hypergeometric enrichment tests, which yielded highly significant *p*‐values (for the normal diet comparison: *pSCx = 9.508e‐175*; for the ketogenic diet effect: *pSCx = 2.668e‐50*,).Subcellular localization analysis via DeepLoc v2.1 (Ødum et al. 2024) further revealed a significant enrichment of mitochondrial proteins in both regions (*pSCx mito = 1.543e‐06*), suggesting enrichment of mitochondrial‐associated proteins under disease and ketogenic diet conditions.

#### 3.3.2 Protein–Protein Interaction and Pathway Enrichment

Enrichment analyses revealed distinct pathway signatures for somatosensory cortex in GAERS versus Wistar comparisons, the top enriched pathways in SCx included metabolism, protein metabolism, axon guidance, nervous system development, amino acid metabolism, cellular responses to stress and chemical stimuli, signaling by ROBO receptors, and regulation of SLITs and ROBOs (Figure 2).

**FIGURE 2:**
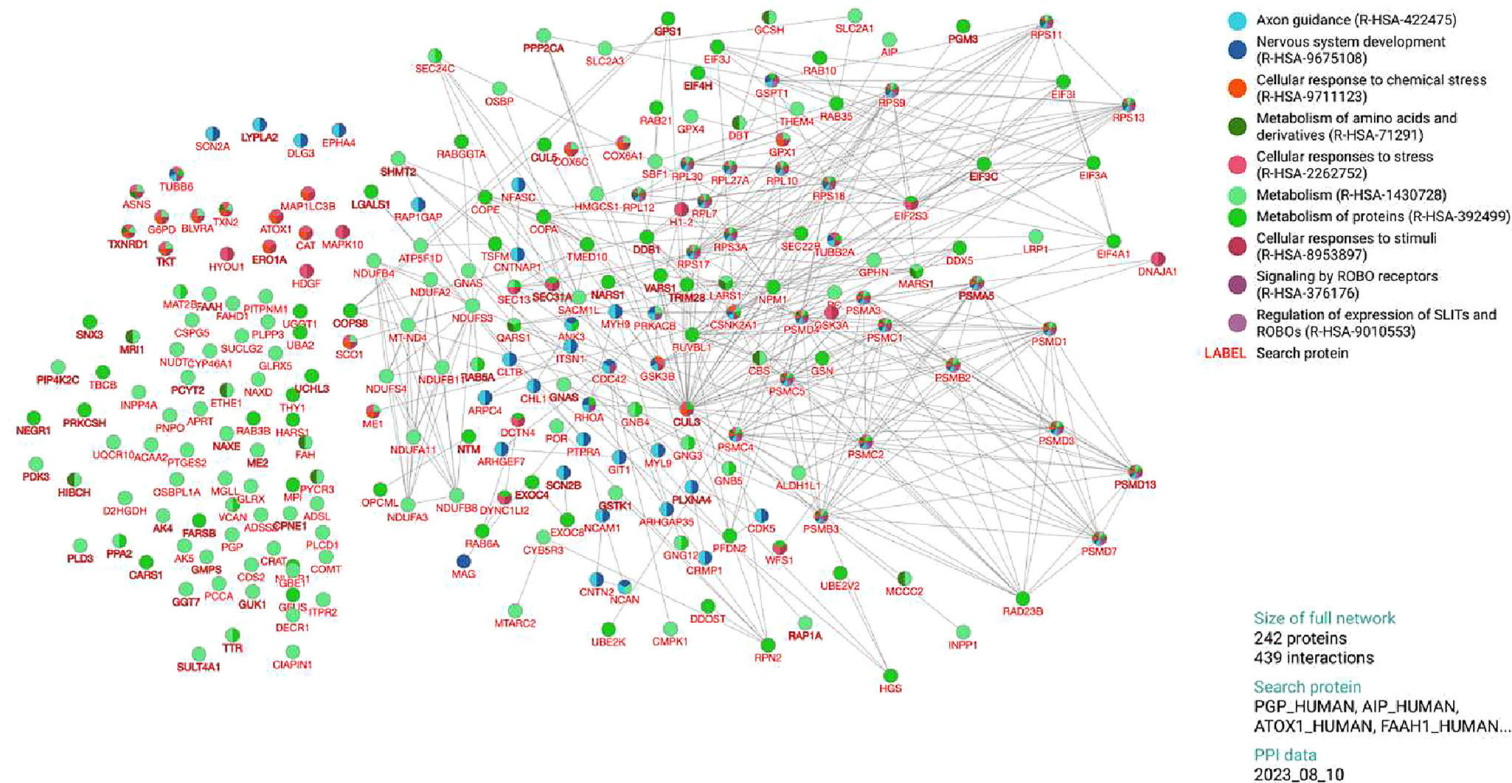
Protein–protein interaction (PPI) network showing proteins associated with the 10 most significantly enriched Reactome pathways identified using ZS‐Revelen in the somatosensory cortex (SCx) of genetic absence epilepsy rats from Strasbourg (GAERS) fed a normal diet compared with Wistar rats fed a normal diet. Node colors indicate the enriched Reactome pathways, and proteins associated with multiple pathways are represented by multicolored nodes.

Following ketogenic diet treatment, enrichment analysis of altered proteins and proteins showing expression shifts toward the Wistar expression profile highlighted several neurodevelopmental and stress‐response pathways. In the SCx, pathways such as axon guidance, L1CAM interactions, trans‐Golgi network vesicle budding, and CRMP signaling in Sema3A were enriched (Figure 3).

**FIGURE 3:**
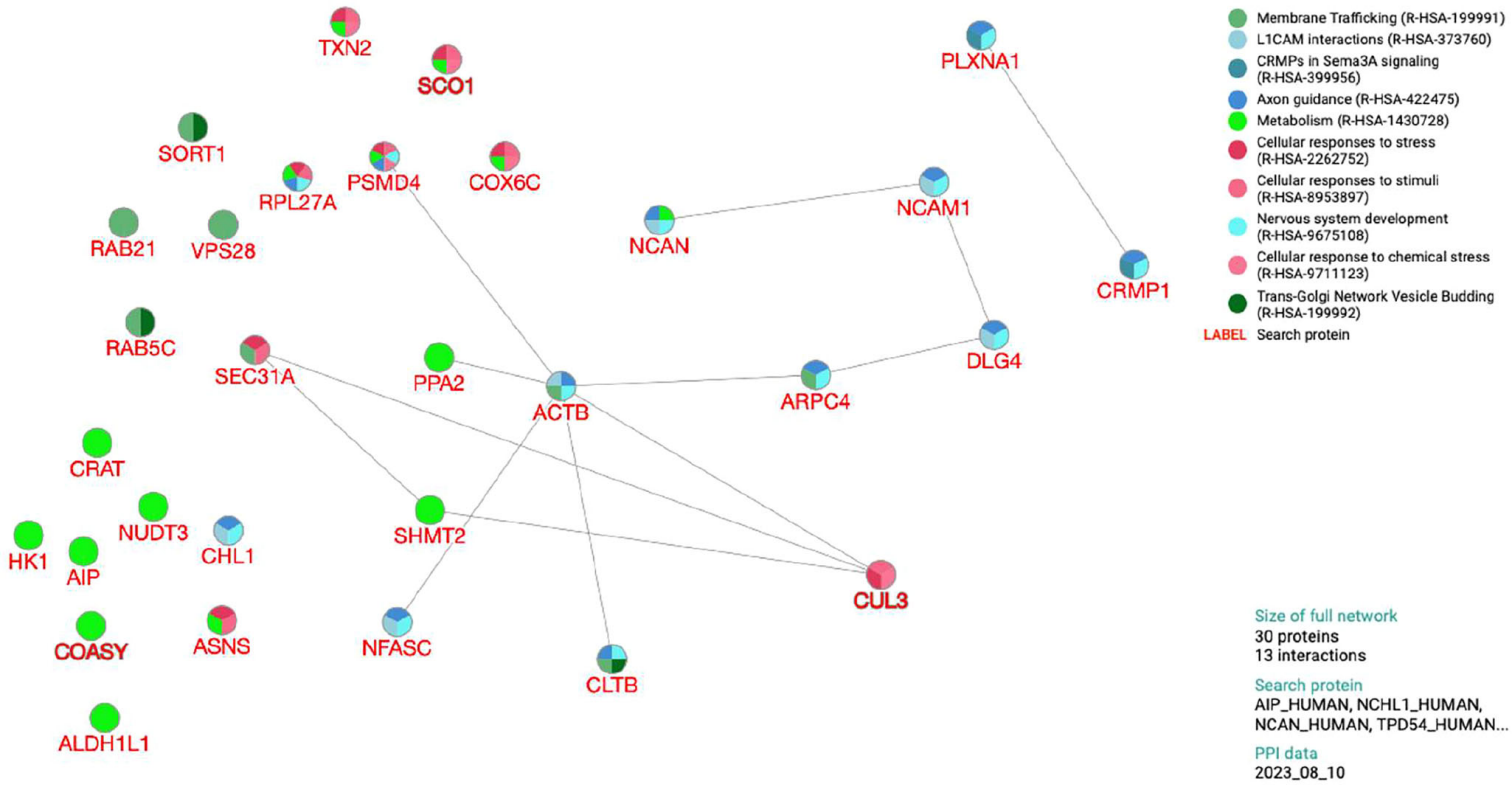
Protein–protein interaction (PPI) network showing proteins associated with the 10 most significantly enriched Reactome pathways identified using ZS‐Revelen in the somatosensory cortex (SCx) of genetic absence epilepsy rats from Strasbourg (GAERS) fed a ketogenic diet compared with GAERS fed a normal diet. The network includes proteins showing expression shifts following ketogenic diet treatment, irrespective of the |log_2_FC| threshold. Node colors indicate the enriched Reactome pathways, and proteins associated with multiple pathways are represented by multicolored nodes.

#### 3.3.3 PCA Analysis

Each group was compared by PCA analysis. Principal component (PC) 1 explained 45.4% of the observed variation and captured the major variance across groups in the cortex. Additional components, including PC4 (8.5%), were also examined, as they provided complementary visualization of group‐specific separation (Figure 4). Although these subsequent components accounted for a smaller fraction of variation, their inclusion improved visualization of group‐specific clustering.

**FIGURE 4:**
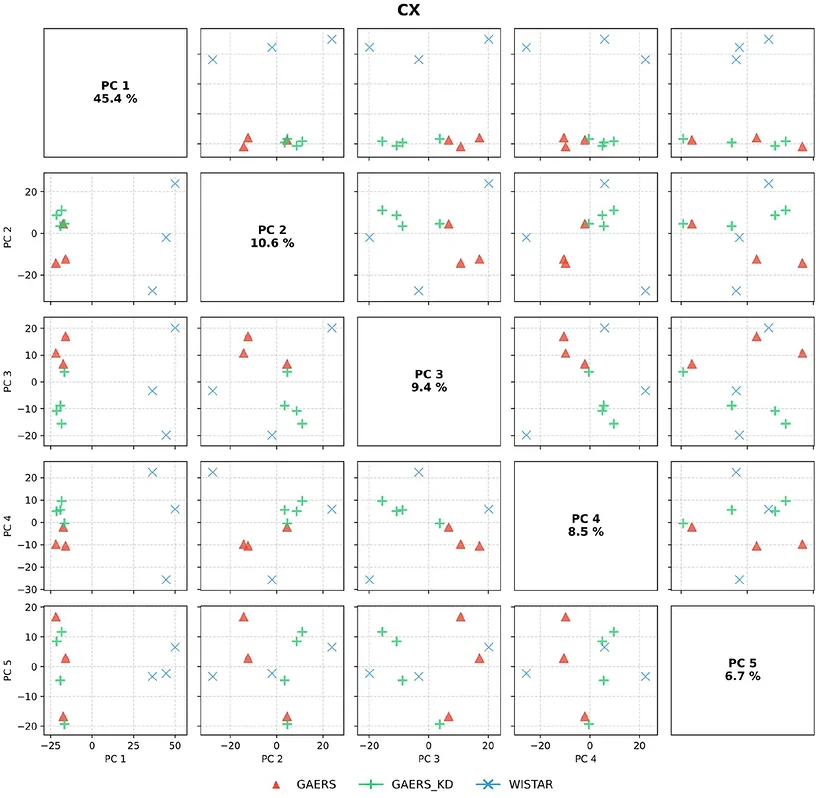
Principal component analysis (PCA) comparing Wistar somatosensory cortex with normal diet (Wis‐Normal diet‐SCx), GAERS somatosensory cortex with normal diet (GA‐Normal diet‐SCx), and GAERS somatosensory cortex with ketogenic diet (GA‐Ketogenic diet‐SCx). Principal component (PC) 1 explained 45.4% of the observed variation and captured the major variance across groups. Additional components, including PC4 (8.5%), were also examined, as they improved the visualization of group‐specific separation.

## 4 Discussion

In this study, we investigated the effect of ketogenic diet on the brain proteome in genetic absence epilepsy rats from Strasbourg. Our findings revealed significant alterations in protein expression profiles in the somatosensory cortex, with a substantial proportion of these changes being synaptic and mitochondrial. It is noteworthy that ketogenic diet administration was associated with alterations in metabolic pathways and shifted the expression of multiple proteins that were dysregulated in GAERS, suggesting potential molecular adaptations. Furthermore, pathway enrichment analyses highlighted key biological processes associated with the KD. In the SCx, these included nervous system development, axon guidance, trans‐Golgi network, membrane trafficking, cellular response mechanisms, and metabolism. Previous studies have suggested that the effects of ketogenic diet are not limited to metabolic regulation but involve coordinated changes in multiple biological processes relevant to neuronal function and seizure control (Boison 2017; K. Bough 2008; K. J. Bough and Rho 2007). The broad range of enriched pathways identified in the present study is consistent with these observations and supports the presence of widespread molecular adaptations in the somatosensory cortex following ketogenic diet treatment.

In the proteomic analysis, proteins showing expression shifts following ketogenic diet treatment were identified. According to the DisGeNET and DOID databases, nine of these proteins (Asns, Crmp1, Ncam1, Nfasc, Sco1, Slc4a10, Sv2b, Tollip, and Txn2) have previously been associated with seizures and epilepsy. The observed expression shifts in these epilepsy‐associated proteins support the possibility that ketogenic diet administration may influence molecular pathways relevant to absence epilepsy.

### 4.1 Prominent Pathways Upon Ketogenic Diet in the SCx

The 10 most prominent pathways identified by ZS Revelen are discussed below.

Axon guidance and nervous system development pathways (encompassing axon guidance, nervous system development, L1CAM interactions, and CRMPs in Sema3A signaling) were the highest ranked pathways. A total of 12 associated proteins in the SCx of GAERS ketogenic diet versus GAERS normal diet were identified: Cltb, Rpl27a, Nfasc, Ncan, Psmd4, Ncam1, Plxna1, Chl1, Arpc4, Dlg4, Actb, and Crmp1. Following ketogenic diet treatment, the expression patterns of Arpc4, Cltb, Dlg4, Actb, Ncam1, Nfasc, Rpl27a, Plxna1, and Chl1 shifted toward those observed in Wistar rats, whereas the expression of Ncan and Psmd4 increased in ketogenic diet‐fed GAERS compared with normal diet‐fed GAERS. Notably, these two proteins already showed more than two log_2_ fold change upregulation in GAERS compared to Wistar rats.

Among these, Plxna1 and Crmp1 function together in the Semaphorin‐3A signaling axis, where Plxna1 serves as the receptor initiating the repulsive guidance signal. Crmp1, as a downstream effector, undergoes phosphorylation and mediates cytoskeletal rearrangements through with Filamin‐A (F. Nakamura et al. 2014). This process is important for proper axonal navigation during development, and altered semaphorin signaling has been associated with synaptic reorganization in experimental epilepsy (Barnes et al. 2003). Notably, GSK3α and GSK3β, which are key kinases known to phosphorylate Crmp1 and inhibit microtubule polymerization (Lin, Chan, Hall, and Manser 2011), were also upregulated more than three fold in GAERS versus Wistar. These findings are consistent with the possibility that Crmp1‐associated signaling may be altered in the epileptic GAERS cortex, potentially contributing to changes in cytoskeletal organization and axonal guidance. Interestingly, while GSK3α/β levels remained unchanged upon ketogenic diet treatment, Crmp1 expression was modestly increased in KD‐fed GAERS compared to GAERS. This may reflect a molecular response associated with ketogenic diet treatment; however, the underlying mechanisms remain to be determined. Among other significantly altered proteins, Nfasc, Chl1, Ncan and Ncam1 are cell adhesion molecules. Elevated Ncan expression has been reported across several experimental epilepsy models, supporting its involvement in epilepsy‐related extracellular matrix remodeling. For example, kainic acid–induced seizures result in increased Ncan expression in limbic brain regions, with sustained upregulation observed during chronic epileptic states (Okamoto et al. 2003). Consistent with these findings, increased Ncan levels have also been confirmed in independent mouse models of epilepsy, including both kainate‐induced and Bassoon knockout models (Blondiaux et al. 2023). In line with this literature, we observed a marked upregulation of Ncan in GAERS compared to Wistar rats. Notably, ketogenic diet treatment was associated with reduced Ncan expression in GAERS, suggesting a potential association with extracellular matrix remodeling in epilepsy.

Ncam1 is a well‐characterized cell adhesion molecule involved in neuronal migration, axon fasciculation, and synapse formation, and plays a critical role in the structural organization and stability of neuronal circuits (Cremer et al. 1997; Dityatev et al. 2004). Dysregulated Ncam1 expression has been implicated in seizure susceptibility and neuronal hyperexcitability, with multiple studies reporting increased expression of several NCAM isoforms across experimental epilepsy models, including kainic acid–induced seizures (Le Gal La Salle et al. 1992). Consistent with these observations, human temporal lobe epilepsy studies have demonstrated upregulation of NCAM in epileptic brain regions, supporting the notion that synaptic remodeling is a persistent feature of epileptic networks (Mikkonen et al. 1998). In line with this, Sato et al. (2002) showed that repeated generalized seizures in the amygdala kindling model result in a marked increase in PSA‐NCAM–positive cells in the dentate gyrus, suggesting that seizure‐induced modulation of NCAM contributes to synaptic reorganization. Furthermore, transgenic mice overexpressing the soluble extracellular domain of NCAM (NCAM‐EC) exhibit a pronounced reduction in synaptic terminals of specific GABAergic interneuron populations in frontal cortical regions and the amygdala, highlighting the importance of balanced NCAM signaling for inhibitory circuit integrity (Pillai‐Nair et al. 2005). In the present study, we observed that ketogenic diet treatment shifted the altered expression of Ncam1 in the somatosensory cortex, which may be associated with molecular changes related to synaptic organization and network adaptation under ketogenic diet administration.

Another important protein is Dlg4 (another name is PSD‐95), which decreased upon ketogenic diet treatment. However, we did not identify a significant change in Dlg4 in the comparison of GAERS versus Wistar; instead, we observed that another synaptic scaffold protein Dlg3 is increased more than two log_2_FC in GAERS compared to Wistar. Dlg3 and Dlg4 are both classified under the membrane‐associated guanylate kinase (MAGUK) family of proteins (Gardoni, Marcello, and Di Luca 2009). Among these, Dlg4—commonly known as PSD‐95—serves as a critical scaffolding molecule within the postsynaptic density (PSD), where it is predominantly found at excitatory glutamatergic synapses. Through its direct binding to stargazin, PSD‐95 facilitates the trafficking and synaptic integration of AMPA receptors (Schnell et al. 2002). Dlg4 gene variants can cause Dlg4‐related synaptopathy, a neurodevelopmental disorder with developmental delay, intellectual disability, and often epilepsy (Rodríguez‐Palmero et al. 2021). A study of 35 individuals with Dlg4 variants revealed epilepsy in approximately 50% of them. Over a quarter of these individuals presented with developmental epileptic encephalopathy with spike‐wave activation during sleep or status epilepticus associated with spike‐wave activation during sleep (Kassabian et al. 2024). In adolescence, PSD‐95 haploinsufficiency (PSD‐95+/−) altered GABA_A_ receptor expression and function in the medial prefrontal cortex (mPFC), with a notable upregulation of the α1 subunit. Corresponding electrophysiological changes included increased frequency and amplitude of spontaneous inhibitory postsynaptic currents (sIPSCs) and significantly enhanced evoked IPSCs in pyramidal neurons of the mPFC in PSD‐95+/− mice (McEachern et al. 2020). This observation is consistent with our finding of decreased PSD‐95 expression in ketogenic diet–treated GAERS and may be associated with changes in synaptic organization under ketogenic diet treatment.

When we looked at the metabolism associated proteins upon ketogenic diet, a number of proteins were enriched in this pathway, including Txn2, Psmd4, Cox6c2, Ncan, Rpl27a, Sco1, Ppa2, Aip, Nudt3, Crat, Shmt2, Coasy, Aldh1l1 and Asns. The expression direction of Txn2, Ncan, Rpl27a, Sco1, Coasy, Aldh1l1, and Asns shifted following ketogenic diet application. Others (Psmd4, Cox6c2, Ppa2, Aip, Nudt3, Crat, and Shmt2) are increased in the GAERS fed with ketogenic diet compared to GAERS but the increment in this group is not high compared to GAERS versus Wistar comparison. Cox6c2 and Sco are mitochondrial proteins involved in the assembly of cytochrome c oxidase, a critical component of the electron transport chain. Therefore, they play a role in energy production. Other players in central carbon metabolism and energy production are Ppa2, (inorganic pyrophosphatase, which catalyzes the hydrolysis of pyrophosphate to inorganic phosphate) and Crat (carnitine O‐acetyltransferase, involved in fatty acid metabolism, specifically carnitine shuttle). Shmt2 (serine hydroxymethyltransferase 2) is a key enzyme in one‐carbon metabolism, converting serine to glycine and providing one‐carbon units for nucleotide synthesis. Moreover, it is involved in the reversible synthesis of serine and glycine from each other. If we look at the electron transfer chain and energy metabolism related proteins, we have to consider the other proteins that had their expression level changed moderately with ketogenic diet. The expression of Mt‐nd4, Sco1, Tomm34, Dld, Hk1, and Txn2 was decreased, while that of Cox6c2, Crat, Acaa1, Eci2, Cox2, Aco1, Acat1, Bcat1, and Hadha (Table 1) was increased in ketogenic diet‐treated GAERS Scx. The observed protein expression profile suggests metabolic adaptation in epileptic cortex following ketogenic diet treatment and is consistent with molecular changes associated with increased reliance on ketone utilization over glucose. Expression of proteins associated with mitochondrial complex I (e.g., Mt‐nd4) and pyruvate oxidation (Dld) were reduced, along with mitochondrial import component Tomm34. Conversely, proteins involved in fatty acid β‐oxidation and ketone body metabolism (Crat, Acaa1b, Eci2, Aco1, Acat1, Hadha, and Bcat1) were upregulated, which is consistent with increased utilization of lipid‐derived energy reported in ketogenic diet studies (K. J. Bough et al. 2006). Upregulation of Cox2 and Cox6c2—components of complex IV—may be associated with mitochondrial adaptation to ketogenic diet treatment. Meanwhile, decreased Txn2 expression may be associated with alterations in mitochondrial redox homeostasis. Altogether, these findings suggest that ketogenic diet administration is associated with mitochondrial and metabolic adaptations in the epileptic cortex (K. J. Bough et al. 2006).

Sco1, Sec31a, Asns, Cox6c2, Rpl27a, Txn2, and Cul3 are associated with cellular response mechanisms; all proteins except Sec31a and Cox6c2 showed shifts in expression following ketogenic diet treatment. In our study, Asparagine synthetase (Asns) expression was found to be elevated in the GAERS model of absence epilepsy compared to control animals, while ketogenic diet treatment shifted this expression pattern. Although Asns deficiency has been linked to severe epileptic encephalopathies caused by biallelic loss‐of‐function mutations in the Asns gene (Ben‐Salem et al. 2015; Liu et al. 2022; Ruzzo et al. 2013), our findings suggest a distinct, nongenetic, and metabolic adaptation. Asns catalyzes the ATP‐dependent conversion of aspartate and glutamine into asparagine and glutamate (Tesson et al. 2003). In the brain, glutamine functions as a key precursor for synaptic glutamate synthesis via the glutamate‐glutamine cycle, particularly at excitatory presynaptic terminals. Thus, increased Asns expression in epileptic animals may be associated with alterations in glutamine–glutamate metabolism, which could influence excitatory neurotransmission. Conversely, decreased Asns expression following ketogenic diet treatment may reflect metabolic adaptation and may be associated with alterations in glutamine–glutamate metabolism, potentially contributing to molecular responses associated with ketogenic diet administration.

When proteins showing expression shifts following ketogenic diet treatment were analyzed using enrichment tools, membrane trafficking emerged as a prominent associated pathway. Membrane trafficking pathways are crucial for cellular homeostasis, and their dysregulation has been implicated in neurological disorders, including epilepsy. Key regulators of vesicle‐mediated transport, influencing intracellular trafficking and signaling, include proteins such as Arpc4, Rab21, Cltb, and Vps28. Our study indicates that, in GAERS rats compared to Wistar rats, Arpc4, Rab21, and Vps28 exhibit increased expression, whereas Cltb shows reduced expression. Interestingly, ketogenic diet treatment shifted these expression patterns, suggesting that proteins involved in membrane trafficking may contribute to molecular adaptations in the cortex under ketogenic diet treatment.

Rab21, a member of the Rab GTPase family, regulates endosomal trafficking, including the trafficking of β1‐integrins (Pellinen et al. 2006). Dysregulation of Rab proteins has been linked to neuroinflammation and synaptic dysfunction. Arpc4, a component of the actin‐related protein complex, plays a role in cytoskeletal remodeling, which is essential for vesicle movement and synaptic plasticity. Arpc4 is subunit of the Arp2/3 complex, and is known to play a critical role in actin polymerization and remodeling of neuronal structure (Goley et al. 2010). Although its direct association with epilepsy and related mechanisms are not known, the pathogenic variants in the Arpc4 gene have been reported to cause neurodevelopmental disorders such as microcephaly, speech delay, and neurodevelopmental disorder with mild intellectual disability, epilepsy, and growth retardation (Jayaraman et al. 2018; Kuroda et al. 2023; Laboy Cintron et al. 2022). Vps28, part of the ESCRT1 machinery, is involved in sorting ubiquitinated proteins for degradation. Alterations in ESCRT components have been associated with neurodegenerative conditions. ESCRT components play critical regulatory roles in neuron morphology and ESCRT protein deficiency influences dendritic morphology by modulating the mTOR signaling pathway. Reduced ESCRT activity has been associated with lower mTOR pathway activation, leading to altered neuronal development and synaptic function (Firkowska et al. 2019). In the somatosensory cortex of GAERS rats compared to Wistar rats, increased expression of Vps28, a key component of the ESCRT‐I complex, may be associated with molecular changes in pathways related to mTOR signaling. Given the critical role of ESCRT proteins in cellular homeostasis, this upregulation may be associated with alterations in vesicular trafficking and protein degradation pathways. Notably, ketogenic diet treatment was associated with reduced Vps28 expression, which may be associated with molecular changes in pathways related to mTOR signaling. Cltb, a clathrin light chain protein, is involved in endocytosis and synaptic vesicle recycling (Poupon et al. 2008; Redlingshöfer et al. 2020). It is essential for endocytosis and synaptic vesicle recycling. Its expression changes in neurological diseases such as Alzheimer's disease; while its expression decreases in Alzheimer's disease patients (Y. Nakamura et al. 1994), it increases in an Alzheimer's disease mouse model (Takano et al. 2013). When epilepsy models were examined, in contrast to our study, the expression of clathrin light chain was increased in models such as electroconvulsive seizure model (Enomoto et al. 2016) and WAG/Rij model of absence epilepsy (Gürol et al. 2015). Together, these findings suggest that ketogenic diet administration may influence intracellular signaling and membrane trafficking pathways associated with cortical molecular adaptation in epilepsy.

## 5 Conclusion

In conclusion, our proteomic analyses indicate that ketogenic diet administration is associated with broad molecular alterations in the GAERS somatosensory cortex. The observed expression shifts in epilepsy‐associated proteins and changes in pathways related to synaptic function, mitochondrial energy metabolism, and cellular homeostasis provide system‐level insight into cortical molecular responses under ketogenic diet conditions. These findings suggest that ketogenic diet administration may be associated with molecular changes in biological pathways relevant to genetic absence epilepsy and support further investigation of the molecular effects of dietary interventions.

## 6 Limitations

The limitations of this study include that thalamic tissue was not examined, and female rats were not included in the study. One of the limitations of our study is the limited number of animals included in the proteomic analyses; therefore, the findings should be interpreted as exploratory and hypothesis‐generating. A ketogenic diet‐treated Wistar control group was also not included. Accordingly, the present study was designed to evaluate the molecular effects of ketogenic diet treatment within the GAERS model of absence epilepsy rather than its independent effects in the healthy brain. The proteins and pathways identified in this study should therefore be considered as candidate findings requiring further validation. Additional targeted validation and functional studies are needed to confirm these observations and better understand their roles in the molecular effects of ketogenic diet treatment (Jiang et al. 2024; Sobsey et al. 2020).

## Author Contributions

F.O. and A.G. contributed to the study conception and design. F.O., A.G., A.T., and N.B.A. carried out material preparation, F.O., A.G., U.G., B.S., B.O., O.U.S., and A.M. performed data collection and analysis. The first draft of the manuscript was written by F. O. and A.G. and all authors commented on previous versions of the manuscript. All authors read and approved the final manuscript.

## Funding

The authors have nothing to report.

## Conflicts of Interest

The authors declare no conflicts of interest.

## Supporting information




**Table S1**: Differentially expressed proteins in the cortex of GAERS with Normal Diet compared to Wistar normal diet.

**Table S2**: Comparison of differently expressed proteins in the somatosensorial cortex tissue between ketogenic diet‐fed and normal diet‐fed GAERS

**Table S3**: Common proteins between GAERS vs. WISTAR and GAERS_Ketogenic diet_ vs. GAERS_Normal diet_

## Data Availability

The mass spectrometry proteomics data have been deposited to the ProteomeXchange Consortium via the PRIDE partner repository with the dataset identifier PXD081865 (Perez‐Riverol et al., 2022).

## References

[brb371757-bib-0001] Acikgoz, N. B. , A. Toplu‐Hocaoglu , B. A. Yildirim , H. R. Yananli , D. Turkdogan , and F. Onat . 2026. “Effect of the Ketogenic Diet on Absence Seizures in Rats With Genetic Absence Epilepsy.” Epilepsy Research 226: 107847. 10.1016/j.eplepsyres.2026.107847. 42341707

[brb371757-bib-0002] Aebersold, R. , J. N. Agar , I. J. Amster , et al. 2018. “How Many Human Proteoforms are There?.” Nature Chemical Biology 14, no. 3: 206–214. 10.1038/nchembio.2576. PMC5837046 29443976

[brb371757-bib-0003] Barnes, G. , R. S. Puranam , Y. Luo , and J. O. McNamara . 2003. “Temporal Specific Patterns of Semaphorin Gene Expression in Rat Brain After Kainic Acid‐Induced Status Epilepticus.” Hippocampus 13, no. 1: 1–20. 10.1002/hipo.10041. 12625453

[brb371757-bib-0004] Benjamini, Y. , and Y. Hochberg . 1995. “Controlling the False Discovery Rate: A Practical and Powerful Approach to Multiple Testing.” Journal of the Royal Statistical Society: Series B (Methodological) 57, no. 1: 289–300. 10.1111/j.2517-6161.1995.tb02031.x.

[brb371757-bib-0005] Ben‐Salem, S. , J. G. Gleeson , A. M. Al‐Shamsi , et al. 2015. “Asparagine Synthetase Deficiency Detected by Whole Exome Sequencing Causes Congenital Microcephaly, Epileptic Encephalopathy and Psychomotor Delay.” Metabolic Brain Disease 30, no. 3: 687–694. 10.1007/s11011-014-9618-0. PMC4915861 25227173

[brb371757-bib-0006] Blondiaux, A. , S. Jia , A. Annamneedi , et al. 2023. “Linking Epileptic Phenotypes and Neural Extracellular Matrix Remodeling Signatures in Mouse Models of Epilepsy.” Neurobiology of Disease 188: 106324. 10.1016/j.nbd.2023.106324. 37838005

[brb371757-bib-0007] Boison, D. 2017. “New Insights into the Mechanisms of the Ketogenic Diet.” Current Opinion in Neurology 30, no. 2: 187–192. 10.1097/wco.0000000000000432. PMC5409832 28141738

[brb371757-bib-0008] Bough, K. 2008. “Energy Metabolism as Part of the Anticonvulsant Mechanism of the Ketogenic Diet.” Supplement, Epilepsia 49, no. S8: 91–93. 10.1111/j.1528-1167.2008.01846.x. PMC3056236 19049599

[brb371757-bib-0009] Bough, K. J. , and J. M. Rho . 2007. “Anticonvulsant Mechanisms of the Ketogenic Diet.” Epilepsia 48, no. 1: 43–58. 10.1111/j.1528-1167.2007.00915.x. 17241207

[brb371757-bib-0010] Bough, K. J. , J. Wetherington , B. Hassel , et al. 2006. “Mitochondrial Biogenesis in the Anticonvulsant Mechanism of the Ketogenic Diet.” Annals of Neurology 60, no. 2: 223–235. 10.1002/ana.20899. 16807920

[brb371757-bib-0011] Cox, J. , and M. Mann . 2008. “MaxQuant Enables High Peptide Identification Rates, Individualized p.p.b.‐Range Mass Accuracies and Proteome‐Wide Protein Quantification.” Nature Biotechnology 26, no. 12: 1367–1372. 10.1038/nbt.1511. 19029910

[brb371757-bib-0012] Cox, J. , N. Neuhauser , A. Michalski , R. A. Scheltema , J. V. Olsen , and M. Mann . 2011. “Andromeda: A Peptide Search Engine Integrated into the MaxQuant Environment.” Journal of Proteome Research 10, no. 4: 1794–1805. 10.1021/pr101065j. 21254760

[brb371757-bib-0013] Cremer, H. , G. Chazal , C. Goridis , and A. Represa . 1997. “NCAM is Essential for Axonal Growth and Fasciculation in the Hippocampus.” Molecular and Cellular Neuroscience 8, no. 5: 323–335. 10.1006/mcne.1996.0588. 9073395

[brb371757-bib-0014] D'Arcangelo, G. , M. D'Antuono , G. Biagini , R. Warren , V. Tancredi , and M. Avoli . 2002. “Thalamocortical Oscillations in a Genetic Model of Absence Seizures.” European Journal of Neuroscience 16, no. 12: 2383–2393. 10.1046/j.1460-9568.2002.02411.x 12492433

[brb371757-bib-0015] Depaulis, A. , O. David , and S. Charpier . 2016. “The Genetic Absence Epilepsy Rat From Strasbourg as a Model to Decipher the Neuronal and Network Mechanisms of Generalized Idiopathic Epilepsies.” Journal of Neuroscience Methods 260: 159–174. 10.1016/j.jneumeth.2015.05.022. 26068173

[brb371757-bib-0016] Dityatev, A. , G. Dityateva , V. Sytnyk , et al. 2004. “Polysialylated Neural Cell Adhesion Molecule Promotes Remodeling and Formation of Hippocampal Synapses.” The Journal of Neuroscience 24, no. 42: 9372–9382. 10.1523/jneurosci.1702-04.2004. PMC6730092 15496673

[brb371757-bib-0017] Enomoto, S. , K. Shimizu , M. Nibuya , et al. 2016. “Increased Expression of Endocytosis‐Related Proteins in Rat Hippocampus Following 10‐Day Electroconvulsive Seizure Treatment.” Neuroscience Letters 624: 85–91. 10.1016/j.neulet.2016.05.015. 27177725

[brb371757-bib-0018] Firkowska, M. , M. Macias , and J. Jaworski . 2019. “ESCRT Proteins Control the Dendritic Morphology of Developing and Mature Hippocampal Neurons.” Molecular Neurobiology 56, no. 7: 4866–4879. 10.1007/s12035-018-1418-9. PMC6647414 30406428

[brb371757-bib-0019] Gardoni, F. , E. Marcello , and M. Di Luca . 2009. “Postsynaptic Density–Membrane Associated Guanylate Kinase Proteins (PSD–MAGUKs) and Their Role in CNS Disorders.” Neuroscience 158, no. 1: 324–333. 10.1016/j.neuroscience.2008.07.068. 18773944

[brb371757-bib-0020] Goley, E. D. , A. Rammohan , E. A. Znameroski , E. N. Firat‐Karalar , D. Sept , and M. D. Welch . 2010. “An Actin‐Filament‐Binding Interface on the Arp2/3 Complex is Critical for Nucleation and Branch Stability.” Proceedings of the National Academy of Sciences 107, no. 18: 8159–8164. 10.1073/pnas.0911668107 PMC2889539 20404198

[brb371757-bib-0021] Groomes, L. B. , P. L. Pyzik , Z. Turner , J. L. Dorward , V. H. Goode , and E. H. Kossoff . 2011. “Do Patients With Absence Epilepsy Respond to Ketogenic Diets?.” Journal of Child Neurology 26, no. 2: 160–165. 10.1177/0883073810376443. 20647578

[brb371757-bib-0022] Guelpa, G. 1911. “La Lutte Contre L'epiepsie Par la Desintoxication et Par la Reeducation Alimentaire.” Revue de Therapeutique Medico‐Chirurgicale 78: 8–13.

[brb371757-bib-0023] Gürol, G. , D. Demiralp , A. K. Yılmaz , Ö. Akman , N. Ateş , and A. Karson . 2015. “Comparative Proteomic Approach in Rat Model of Absence Epilepsy.” Journal of Molecular Neuroscience 55, no. 3: 632–643. 10.1007/s12031-014-0402-8. 25323782

[brb371757-bib-0024] Jayaraman, D. , B.‐I. Bae , and C. A. Walsh . 2018. “The Genetics of Primary Microcephaly.” Annual Review of Genomics and Human Genetics 19: 177–200. 10.1146/annurev-genom-083117-021441. 29799801

[brb371757-bib-0025] Jiang, Y. , D. A. B. Rex , D. Schuster , et al. 2024. “Comprehensive Overview of Bottom‐Up Proteomics Using Mass Spectrometry.” ACS Measurement Science Au 4, no. 4: 338–417. 10.1021/acsmeasuresciau.3c00068. PMC11348894 39193565

[brb371757-bib-0026] Kassabian, B. , A. M. Levy , E. Gardella , et al. 2024. “Developmental Epileptic Encephalopathy in DLG4‐Related Synaptopathy.” Epilepsia 65, no. 4: 1029–1045. 10.1111/epi.17876. 38135915

[brb371757-bib-0027] Koopmans, F. , P. van Nierop , M. Andres‐Alonso , et al. 2019. “SynGO: An Evidence‐Based, Expert‐Curated Knowledge Base for the Synapse.” Neuron 103, no. 2: 217–234. 10.1016/j.neuron.2019.05.002. PMC6764089 31171447

[brb371757-bib-0028] Kuroda, Y. , T. Kumaki , Y. Saito , et al. 2023. “A Novel Variant of ARPC4‐Related Neurodevelopmental Disorder.” American Journal of Medical Genetics Part A 191, no. 3: 893–895. 10.1002/ajmg.a.63082. 36513617

[brb371757-bib-0029] Laboy Cintron, D. , A. M. Muir , A. Scott , et al. 2022. “A Recurrent, De Novo Pathogenic Variant in ARPC4 Disrupts Actin Filament Formation and Causes Microcephaly and Speech Delay.” HGG Advances 3, no. 1: 100072. 10.1016/j.xhgg.2021.100072. PMC8756495 35047857

[brb371757-bib-0030] Le Gal La Salle, G. , G. Rougon , and A. Valin . 1992. “The Embryonic Form of Neural Cell Surface Molecule (E‐NCAM) in the Rat Hippocampus and Its Reexpression on Glial Cells Following Kainic Acid‐Induced Status Epilepticus.” Journal of Neuroscience 12, no. 3: 872–882. 10.1523/JNEUROSCI.12-03-00872.1992. PMC6576067 1545243

[brb371757-bib-0031] Leitner, D. F. , Y. Siu , A. Korman , et al. 2023. “Metabolomic, Proteomic, and Transcriptomic Changes in Adults With Epilepsy on Modified Atkins Diet.” Epilepsia 64, no. 4: 1046–1060. 10.1111/epi.17540. PMC10372873 36775798

[brb371757-bib-0032] Li, T. , R. Wernersson , R. B. Hansen , et al. 2017. “A Scored Human Protein‐Protein Interaction Network to Catalyze Genomic Interpretation.” Nature Methods 14, no. 1: 61–64. 10.1038/nmeth.4083. PMC5839635 27892958

[brb371757-bib-0033] Lin, P.‐C. , P. M. Chan , C. Hall , and E. Manser . 2011. “Collapsin Response Mediator Proteins (CRMPs) are a New Class of Microtubule‐Associated Protein (MAP) That Selectively Interacts With Assembled Microtubules via a Taxol‐Sensitive Binding Interaction.” Journal of Biological Chemistry 286, no. 48: 41466–41478. 10.1074/jbc.M111.283580. PMC3308858 21953449

[brb371757-bib-0034] Liu, L. , J. Wang , H. Li , et al. 2022. “An Intractable Epilepsy Phenotype of ASNS Novel Mutation in Two Patients With Asparagine Synthetase Deficiency.” Clinica Chimica Acta 531: 331–336. 10.1016/j.cca.2022.04.989. 35469797

[brb371757-bib-0035] McEachern, E. P. , A. A. Coley , S. S. Yang , and W. J. Gao . 2020. “PSD‐95 Deficiency Alters GABAergic Inhibition in the Prefrontal Cortex.” Neuropharmacology 179: 108277. 10.1016/j.neuropharm.2020.108277. PMC7572776 32818520

[brb371757-bib-0036] Mikkonen, M. , H. Soininen , R. Kälviänen , et al. 1998. “Remodeling of Neuronal Circuitries in Human Temporal Lobe Epilepsy: Increased Expression of Highly Polysialylated Neural Cell Adhesion Molecule in the Hippocampus and the Entorhinal Cortex.” Annals of Neurology 44, no. 6: 923–934. 10.1002/ana.410440611. 9851437

[brb371757-bib-0037] Nakamura, F. , K. Kumeta , T. Hida , et al. 2014. “Amino‐ and Carboxyl‐Terminal Domains of Filamin‐A Interact With CRMP1 to Mediate Sema3A Signalling.” Nature Communications 5, no. 1: 5325. 10.1038/ncomms6325. 25358863

[brb371757-bib-0038] Nakamura, Y. , M. Takeda , K. Yoshimi , et al. 1994. “Involvement of Clathrin Light Chains in the Pathology of Alzheimer's Disease.” Acta Neuropathologica 87: 23–31. 10.1007/BF00386251 8140893

[brb371757-bib-0039] Ødum, M. T. , F. Teufel , V. Thumuluri , et al. 2024. “DeepLoc 2.1: Multi‐Label Membrane Protein Type Prediction Using Protein Language Models.” Nucleic Acids Research 52, no. W1: W215–W220. 10.1093/nar/gkae237. PMC11223819 38587188

[brb371757-bib-0040] Okamoto, M. , J. Sakiyama , S. Mori , et al. 2003. “Kainic Acid‐Induced Convulsions Cause Prolonged Changes in the Chondroitin Sulfate Proteoglycans Neurocan and Phosphacan in the Limbic Structures.” Experimental Neurology 184, no. 1: 179–195. 10.1016/s0014-4886(03)00251-6 14637091

[brb371757-bib-0041] Pellinen, T. , A. Arjonen , K. Vuoriluoto , K. Kallio , J. A. Fransen , and J. Ivaska . 2006. “Small GTPase Rab21 Regulates Cell Adhesion and Controls Endosomal Traffic of Beta1‐Integrins.” Journal of Cell Biology 173, no. 5: 767–780. 10.1083/jcb.200509019. PMC2063892 16754960

[brb371757-bib-0064] Percie du Sert, N. , A. Ahluwalia , S. Alam , et al. 2020. “Reporting animal research: Explanation and elaboration for the ARRIVE guidelines 2.0.” PLOS Biology 18, no. 7: e3000411. 10.1371/journal.pbio.3000411. PMC7360025 32663221

[brb371757-bib-0042] Perez‐Riverol, Y, J. Bai , C. Bandla , et al. 2022. “The PRIDE Database Resources in 2022: A Hub for Mass Spectrometry‐Based Proteomics Evidences.” Nucleic Acids Research 50, no. D1: D543–D552. 10.1093/nar/gkab1038. PMC8728295 34723319

[brb371757-bib-0043] Pillai‐Nair, N. , A. K. Panicker , R. M. Rodriguiz , et al. 2005. “Neural Cell Adhesion Molecule‐Secreting Transgenic Mice Display Abnormalities in GABAergic Interneurons and Alterations in Behavior.” Journal of Neuroscience 25, no. 18: 4659–4671. 10.1523/jneurosci.0565-05.2005. PMC6725026 15872114

[brb371757-bib-0044] Pluta, R. , and M. Jablonski . 2011. “The Ketogenic Diet for Epilepsy Therapy in Children: Quo vadis?.” Nutrition 27, no. 5: 615–616. 10.1016/j.nut.2010.12.015. 21463752

[brb371757-bib-0045] Poupon, V. , M. Girard , V. Legendre‐Guillemin , et al. 2008. “Clathrin Light Chains Function in Mannose Phosphate Receptor Trafficking via Regulation of Actin Assembly.” Proceedings of the National Academy of Sciences 105, no. 1: 168–173. 10.1073/pnas.0707269105. PMC2224180 18165318

[brb371757-bib-0046] Redlingshöfer, L. , F. McLeod , Y. Chen , et al. 2020. “Clathrin Light Chain Diversity Regulates Membrane Deformation in Vitro and Synaptic Vesicle Formation in Vivo.” Proceedings of the National Academy of Sciences 117, no. 38: 23527–23538. 10.1073/pnas.2003662117. PMC7519287 32907943

[brb371757-bib-0047] Rodríguez‐Palmero, A. , M. M. Boerrigter , D. Gómez‐Andrés , et al. 2021. “ *DLG4*‐Related Synaptopathy: A New Rare Brain Disorder.” Genetics in Medicine 23, no. 5: 888–899. 10.1038/s41436-020-01075-9. 33597769

[brb371757-bib-0048] Ruzzo, E. K. , J. M. Capo‐Chichi , B. Ben‐Zeev , et al. 2013. “Deficiency of Asparagine Synthetase Causes Congenital Microcephaly and a Progressive Form of Encephalopathy.” Neuron 80, no. 2: 429–441. 10.1016/j.neuron.2013.08.013. PMC3820368 24139043

[brb371757-bib-0049] Saei, A. A. , C. M. Beusch , A. Chernobrovkin , et al. 2019. “ProTargetMiner as a Proteome Signature Library of Anticancer Molecules for Functional Discovery.” Nature Communications 10, no. 1: 5715. 10.1038/s41467-019-13582-8. PMC6915695 31844049

[brb371757-bib-0050] Sato, K. , M. Iwai , I. Nagano , M. Shoji , and K. Abe . 2002. “Changes of Localization of Highly Polysialylated Neural Cell Adhesion Molecule (PSA‐NCAM) in Rat Hippocampus With Exposure to Repeated Kindled Seizures.” Brain Research 946, no. 2: 323–327. 10.1016/S0006-8993(02)02616-1. 12137937

[brb371757-bib-0051] Scheffer, I. E. , S. Berkovic , G. Capovilla , et al. 2017. “ILAE Classification of the Epilepsies: Position Paper of the ILAE Commission for Classification and Terminology.” Epilepsia 58, no. 4: 512–521. 10.1111/epi.13709. PMC5386840 28276062

[brb371757-bib-0052] Schnell, E. , M. Sizemore , S. Karimzadegan , L. Chen , D. S. Bredt , and R. A. Nicoll . 2002. “Direct Interactions Between PSD‐95 and Stargazin Control Synaptic AMPA Receptor Number.” Proceedings of the National Academy of Sciences 99, no. 21: 13902–13907. 10.1073/pnas.172511199. PMC129795 12359873

[brb371757-bib-0053] Scott, R. C. 2016. “Network Science for the Identification of Novel Therapeutic Targets in Epilepsy.” F1000Research 5: 893. 10.12688/f1000research.8214.1. PMC4870991 27239287

[brb371757-bib-0054] Smith, P. K. , R. I. Krohn , G. T. Hermanson , et al. 1985. “Measurement of Protein Using Bicinchoninic Acid.” Analytical Biochemistry 150, no. 1: 76–85. 10.1016/0003-2697(85)90442-7. 3843705

[brb371757-bib-0055] Sobsey, C. A. , S. Ibrahim , V. R. Richard , et al. 2020. “Targeted and Untargeted Proteomics Approaches in Biomarker Development.” Proteomics 20, no. 9: e1900029. 10.1002/pmic.201900029. 31729135

[brb371757-bib-0056] Takano, M. , T. Yamashita , K. Nagano , et al. 2013. “Proteomic Analysis of the Hippocampus in Alzheimer's Disease Model Mice by Using Two‐Dimensional Fluorescence Difference in Gel Electrophoresis.” Neuroscience Letters 534: 85–89. 10.1016/j.neulet.2012.11.010. 23276639

[brb371757-bib-0057] Tesson, A. R. , T. S. Soper , M. Ciustea , and N. G. Richards . 2003. “Revisiting the Steady State Kinetic Mechanism of Glutamine‐Dependent Asparagine Synthetase From Escherichia Coli.” Archives of Biochemistry and Biophysics 413, no. 1: 23–31. 10.1016/s0003-9861(03)00118-8. 12706338

[brb371757-bib-0058] Tyanova, S. , T. Temu , P. Sinitcyn , et al. 2016. “The Perseus Computational Platform for Comprehensive Analysis of (prote)Omics Data.” Nature Methods 13, no. 9: 731–740. 10.1038/nmeth.3901. 27348712

[brb371757-bib-0059] van Oostrum, M. , T. M. Blok , S. L. Giandomenico , et al. 2023. “The Proteomic Landscape of Synaptic Diversity Across Brain Regions and Cell Types.” Cell 186, no. 24: 5411–5427. 10.1016/j.cell.2023.09.028 PMC10686415 37918396

[brb371757-bib-0060] Weiss, N. , S. Hameed , J. M. Fernández‐Fernández , et al. 2012. “A Ca(v)3.2/Syntaxin‐1A Signaling Complex Controls T‐Type Channel Activity and Low‐Threshold Exocytosis.” Journal of Biological Chemistry 287, no. 4: 2810–2818. 10.1074/jbc.M111.290882. PMC3268438 22130660

[brb371757-bib-0061] Wheless, J. W. 2008. “History of the Ketogenic Diet.” Supplement, Epilepsia 49, no. S8: 3–5. 10.1111/j.1528-1167.2008.01821.x. 19049574

[brb371757-bib-0062] Youngson, N. A. , M. J. Morris , and J. W. O. Ballard . 2017. “The Mechanisms Mediating the Antiepileptic Effects of the Ketogenic Diet, and Potential Opportunities for Improvement With Metabolism‐Altering Drugs.” Seizure: The Journal of the British Epilepsy Association 52: 15–19. 10.1016/j.seizure.2017.09.005. 28941398

[brb371757-bib-0063] Zheng, Y. Q. , M. F. Jin , G. H. Suo , Y. J. Wu , Y. X. Sun , and H. Ni . 2020. “Proteomics for Studying the Effects of Ketogenic Diet Against Lithium Chloride/Pilocarpine Induced Epilepsy in Rats.” Frontiers in Neuroscience 14: 562853. 10.3389/fnins.2020.562853. PMC7550537 33132826

